# Innovative Pedicle Screw Insertion with Mixed Reality Technology Improves Insertion Accuracy in Spinal Surgery

**DOI:** 10.3390/s25133939

**Published:** 2025-06-24

**Authors:** Shintaro Obata, Akira Shinohara, Daigo Arimura, Shunsuke Katsumi, Hiroki Wakiya, Mitsuru Saito

**Affiliations:** Department of Orthopedic Surgery, Jikei University School of Medicine, 19-18 Nishi-Shimbashi, Minato-Ku, Tokyo 105-8471, Japan; h20ms-obata@jikei.ac.jp (S.O.); xlink67@jikei.ac.jp (M.S.)

**Keywords:** augmented reality, pedicle screw, navigation system, spinal surgery

## Abstract

The accuracy of pedicle screw insertion in pediatric scoliosis correction surgery using augmented reality technology in combination with a conventional navigation system was evaluated, and its usefulness was verified. A retrospective study of patients who underwent mixed reality technology-assisted posterior scoliosis correction and fixation was conducted. In total, 361 pedicle screws inserted with a mixed reality technology-assisted navigation system were analyzed; 25 pedicle screws (6.9%) showed Rao Classification Grade 1 deviation, whereas 0.83% showed Rao Classification Grade 2.3 deviation, which is a clinical deviation. In terms of the relationship between the rotation of the vertebral body and the deviation of the pedicle screw, the pedicle screw tended to deviate more easily when it was necessary to insert the pedicle screw in a more strongly oblique position due to the rotation of the vertebral body. The results suggest that the pedicle screw insertion accuracy with augmented reality technology may be superior to that with conventional navigation alone in scoliosis correction and fusion surgery for scoliosis in children. This system is expected to become a standard support tool for spine surgery and will contribute to improving the success rate of surgery and reducing the burden on the surgeon.

## 1. Introduction

In recent years, there has been remarkable progress in extended reality (XR) technologies such as virtual reality (VR), augmented reality (AR), and mixed reality (MR), which are opening up new possibilities for applications in the medical field, especially in surgery. VR is suitable for preoperative simulation and education in a completely virtual space, allowing surgeons to grasp the patient’s anatomy from multiple angles. In addition, AR and MR are expected to be tools that support intraoperative navigation and real-time structural recognition by fusing the actual surgical environment with virtual information, thereby improving the accuracy and safety of surgery [1]. In particular, MR has an innovative advantage over conventional technologies in that it achieves interactive integration of the real world and digital information, allowing surgeons to simultaneously overlay on-site visual information and virtual surgical plans. In the field of spinal surgery, screws are inserted into the spine during posterior spinal fusion, but important organs, blood vessels, and nerves are located around them, and anatomically accurate installation is required [2,3]. In the case of spinal deformity, in addition to scoliosis, the vertebral body is deformed and rotated, the pedicle is thin, and the pedicle insertion direction is very different from normal dissection.

**Figure 1:**
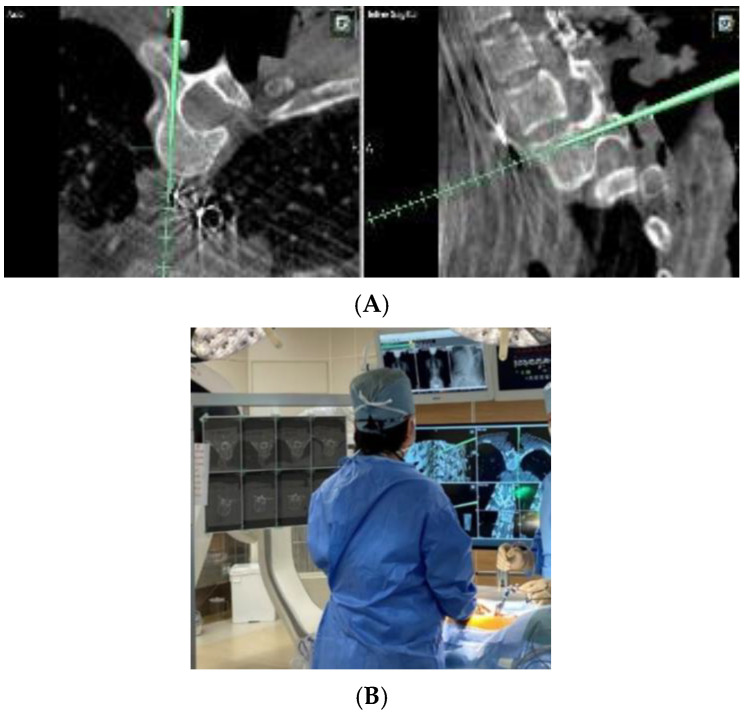
(**A**) Intraoperative navigation image. It is necessary to capture the flat image in three dimensions in the mind and adjust the screw insertion direction. (**B**) When viewing the navigation image, the surgeon takes his or her gaze off the surgical field.

**Figure 2:**
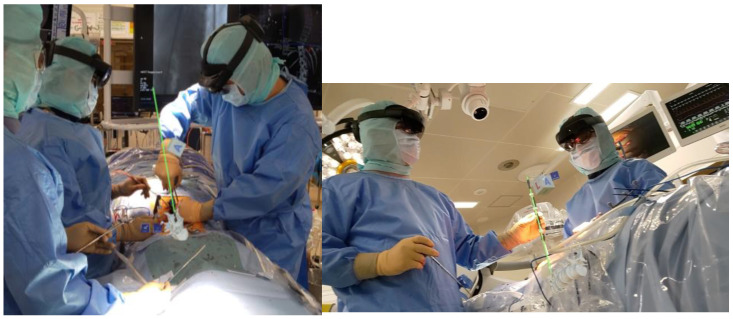
Spinal surgery using virtual lines with augmented reality technical assistance. Although it is used in conjunction with navigation, it is possible to insert a pedicle screw while obtaining information about the surgical field without taking the line of sight off the surgical field.

**Figure 3:**
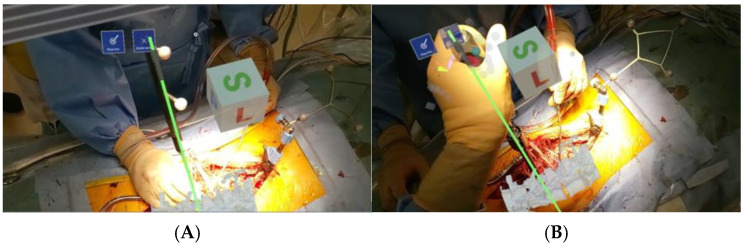
(**A**) Surgeon’s gaze. Navigation guides are matched with virtual lines supported by augmented reality technology. (**B**) A pedicle screw is inserted into the virtual line with 3D augmented reality technical support.

The introduction of a navigation system has been reported to achieve more accurate pedicle screw insertion [4]. The problem with a navigation system is that the navigation screen itself is two-dimensional, but since the surgical field is three-dimensional, it is converted to three dimensions in the surgeon’s head as needed. In addition, since it is necessary to check both the navigation screen and the surgical field, it is problematic to insert the pedicle screw while looking at the navigation screen, removing the line of sight from the surgical field when checking the navigation information (Figure 1). Therefore, we devised a combination of AR technology and inserted a pedicle screw in pediatric scoliosis correction surgery, which is very difficult. In MR, systems using a head-mounted display (HMD) have built-in position detection devices such as infrared sensors and 3D cameras, and they do not require an external camera for alignment. This has the advantage that the surgeon can always check the navigation information at hand within the field of view of the surgical field (Figure 2). Furthermore, in addition to the 3D images acquired before and during surgery, a spatial virtual line function that presents the screw insertion position and direction like a hologram is expected to contribute to improving the accuracy of procedures, improving patient prognosis, and training young surgeons by implementing it in an image support system using HMD (Figure 3). However, it should be noted that MR is only a supplementary image support and cannot be used by itself, because it requires an existing navigation system. In this study, the insertion accuracy of a pedicle screw in combination with a navigation system and MR during surgery was evaluated, and its usefulness in a pediatric spinal deformity case with a thin pedicle and a convoluted vertebral body was verified.

## 2. Materials and Methods

This study was approved by the Ethics Committee of Jikei University School of Medicine, Tokyo. A virtual line guide system using MR technology was applied during pedicle screw insertion under navigation in pediatric scoliosis correction surgery. With the aim of solving the problems of conventional navigation technology, the usefulness of spatial virtual line guidance in spinal surgery was evaluated in actual clinical practice using an MR-guided surgical system with an HMD, and pedicle screw insertion accuracy, superiority, and effects of the combination were examined.

HoloLens 2 (Microsoft Corporation, Redmond, WA, USA) was used as the optical see-through type HMD, and Holoeyes MD (Holoeyes Co., Ltd., Tokyo, Japan) [5], a managed medical device, was used as an application (Figure 1). Holoeyes MD integrates image data obtained from multiple modalities and displays 3D organ models like holograms, which are widely used for preoperative planning and surgical support [6,7,8,9,10,11]. Holoeyes MD is an application that uploads 3D polygon models (STL files) created with DICOM data editing programs such as VINCENT (FUJIFILM Co., Ltd., Tokyo, Japan) from CT data to the Holoeyes server and then downloads them to the terminal at hand, enabling the polygon models to be freely displayed in three dimensions. The application allows the polygon model to be freely moved, zoomed in and out, and displayed in cross-section. The application is included within the Holoeyes MD application. The Virtual Line function allows a line with three-dimensional information to be placed in space, so that the two-dimensional information on the direction of the vertebral root screw insertion confirmed by navigation can be manually aligned by the surgeon and placed as three-dimensional information on the operating field through the Hololens screen.

The accuracy of pedicle screw placement was evaluated in 24 cases (361 screws) of posterior scoliosis correction and fusion surgery for pediatric scoliosis performed at Tokyo Jikei University School of Medicine using MRI and navigation. As a comparison, the results of 21 cases (391 screws) of posterior scoliosis correction and fusion surgery for pediatric scoliosis performed at the same institution using navigation were used.

The patients in the M group/N group included 23 females/18 females and 1 male/3 males, with an average age at surgery of 15.4 years (range: 11–22 years)/16.2 years (range: 12–25 years). The breakdown of cases was as follows: 23/17 cases of adolescent idiopathic scoliosis (Lenke Type 1: 11/7 cases, Type 2: 3/2 cases, Type 5: 9/8 cases) and 1/4 cases of symptomatic scoliosis. Screw insertion was performed by two spinal surgeons.

**Figure 4:**
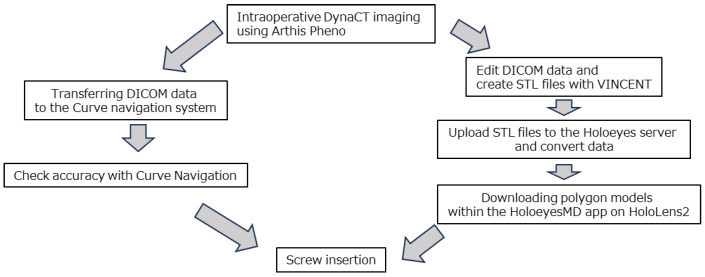
System data flow.

**Figure 5:**
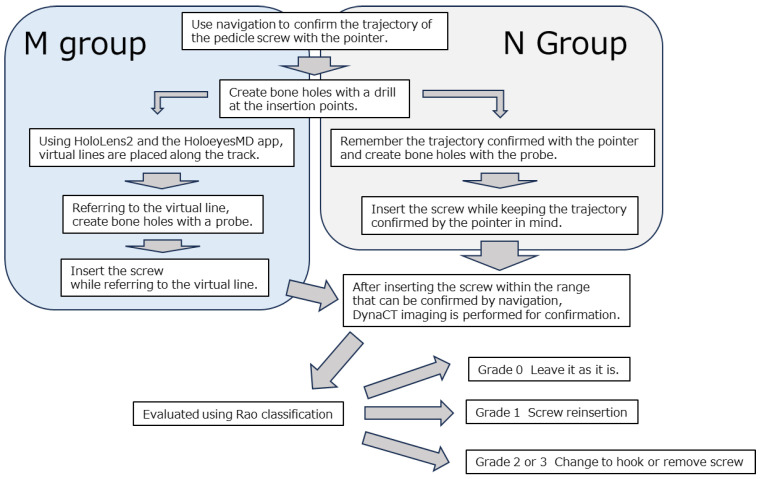
M group and N group workflows.

First, a reference frame was placed on the spinous process under general anesthesia, and DynaCT was performed using ARTIS Pheno (Siemens Healthcare, Forchheim, Germany). The DICOM data were then sent to the navigation system (Brainlab Curve, Brainlab, Westchester, IL, USA) to confirm the accuracy of the navigation. Concurrently, the DICOM data were edited using VINCENT to create an STL file of the spine, which was then uploaded to the Holoeyes server. The file was converted into a polygon model usable in the app and confirmed that the polygon data could be downloaded and used on the HoloeyesMD app on the HoloLens2 (Figure 4). In the M group, the spinal polygon model was displayed to visually assess the rotation of the vertebral bodies, and a virtual line was set on the PS trajectory confirmed by navigation using MR. Pedicle screws were then inserted while confirming the surgical field. In the N group, the surgeon memorized the PS trajectory confirmed by the navigation system and inserted the pedicle screw (Figure 5). In cases where rotation was severe, and it was difficult to visualize the overall picture, a 3D model of the spine was created from CT images taken during surgery, which was then superimposed on the actual surgical field to visualize the rotation of the vertebral body before inserting the screw.

The items to be examined included screw insertion accuracy, direction of deviation, relationship between deviation and vertebral body rotation, and complications. For penetration accuracy, grades 2 and 3 were classified as clinical deviations using the Rao classification [12]. A linear mixed model was used for statistical analysis for comparison, with a significance level of 5%. A pedicle screw was inserted in combination with a virtual line guide system using MR technology for navigation, and it was confirmed that there was no potential drop. After insertion of all pedicle screws, DynaCT imaging was performed. At this time, replacing the screws of Rao classification grade 1, removing the screws of grades 2 and 3 in principle, changing them to hooks and Nespron tapes (Alfresa Pharma Corporation, Osaka, Japan), and re-inserting the pedicle screws to the extent possible for the vertebral bodies that required pedicle screw insertion were considered.

## 3. Results

When using HoloLens 2, the virtual lines of Holoeyes MD were able to merge with the real space within the surgeon’s field of vision, providing the necessary guidance during the procedure. HoloLens 2 can accurately detect the position of the user’s fingers and feed it back to the presented image using an optical position sensor on the forehead, allowing the hologram to rotate, move, enlarge, and change the transparency in the air during surgery, even while wearing sterile gloves. This suggests that it may contribute to reducing surgeons’ stress during surgery. In addition, when checking the monitor screen of the navigation device, the same information could be confirmed in the air of the surgical field, and the movement of the surgeon’s line of sight was minimized.

**Figure 6:**
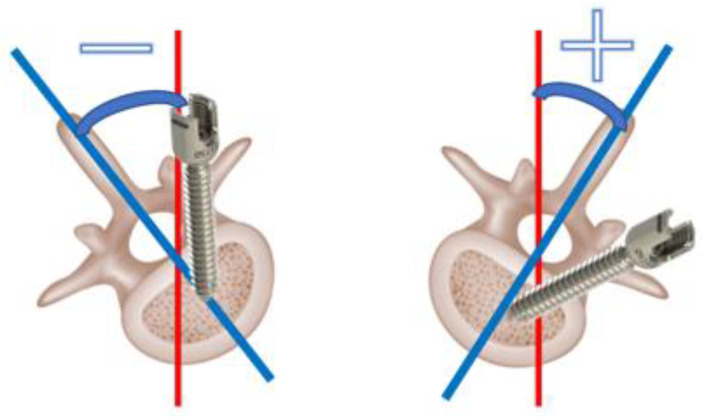
Association of vertebral body rotation and deviation. The angle at which the screw needs to be inserted at a stronger oblique position than usual is positive, and the angle at which it needs to be inserted upright is minus.

The insertion accuracy of pedicle screws in the M group and N group was verified. Deviations classified as Rao grade 1 were observed in 25 screws (6.9%) in the M group and 24 screws (6.1%) in the N group, which were comparable. However, deviations classified as Rao grade 2 were observed in 1 screw (0.3%) in the M group and 11 screws (2.8%) in the N group, and Grade 3 deviations were observed in 2 screws (0.6%) in the M group and 1 screw (0.3%) in the N group. The combined clinical deviation rate for Grades 2 and 3 was 0.83% in the M group and 3.1% in the N group, indicating that the MR group demonstrated significantly higher insertion accuracy (Table 1). In this study, the direction of pedicle screw deviation confirmed by intraoperative C T was all external deviation, and the percentage of lateral deviation on the left and right sides was almost the same. Next, pedicle size was measured by the trabecular bone width at the screw insertion site on the preoperative CT, and pedicle size showed a tendency to be smaller in Grades 2 and 3 than in Grades 0 and 1, with a lower *p* value in Group M than in Group N (Table 2). Next, vertebral rotation was investigated. In the comparison of vertebral rotation between preoperative prone CT (with the patient conscious) and intraoperative prone CT (under anesthesia), there was a tendency toward larger changes in the mid-thoracic and lumbar regions (Table 3). Therefore, the reliability of the screw insertion angle planned based on preoperative CT is not accurate, and evaluation using intraoperative CT imaging is important. Next, the relationship between vertebral rotation and displacement was examined (Figure 6). In the M group, vertebral rotation caused the pedicle screws to deviate strongly at an angle, whereas no such tendency was observed in the N group (Table 4). There were no complications associated with the screw, such as neurological disorders, after surgery.

## 4. Discussion

The preoperative planned screw insertion path was visualized as a hologram as a virtual guide in real time during surgery. Holoeyes MD’s virtual line feature was particularly useful, especially in combination with HoloLens 2. For example, at the time of pedicle screw insertion, it was possible to display it as auxiliary visual information by superimposing it on the surgical field. High-precision 3D polygonal models of the spine, skin, and blood vessels were generated from medical images such as CT and MRI, and the optimal route for the procedure was designed to display the safest and most efficient routes as virtual lines, taking into account anatomical landmarks and important structures (Figure 3). In combination with a conventional navigation system, the information on the monitor screen was superimposed in the air above the surgical field, making it possible to perform surgery without taking the line of sight off the surgical field.

Creating and making a 3D polygon model usable takes at least 10 min, and more time if it needs to be more detailed. However, a simple model can be made usable in about 15 min. Performing this task during surgery may interfere with the surgery, so support from a doctor who is not in the surgical field is necessary. However, utilizing the virtual line requires a certain technique to set it in line with the navigation system pointer, and although it may take 2–3 min for those who are not familiar with it, once mastered, it can be set in about 10 s, and with the cooperation of an assistant, it can be performed without interfering with the surgery. In addition, to prevent the polygon from overlapping with the target object and becoming difficult to identify, the contrast is set low so that both can be identified to a certain extent. In addition, to make it easier to perceive depth, virtual lines are set to be thin, contrast is set to be low, and information about three-dimensionality can be obtained by checking from multiple directions [13]. It should be noted that these are for the surgeon to visually confirm and manually verify, and they do not guarantee accuracy themselves, but merely add to the information that can be confirmed during surgery.

The challenge with navigation systems is that they require the surgeon to take his or her eyes off the operative field and look at an external monitor, as well as the need for the surgeon to transform two-dimensional information into three-dimensional information. In this study, the aim is for the surgeon to obtain three-dimensional vertebral deformation and screw insertion direction information without taking his eyes off the operating field by using polygons and virtual lines displayed in the operating field on the basis of the accurate navigation information displayed on the monitor. Mixed Reality Capture and other technologies sometimes display virtual objects by synthesizing them in 2D from recorded video, but the HoloLens 2 used in this study uses multiple sensors, including depth sensors, inside-out tracking cameras, accelerometers, gyroscopes, magnetometers, and RGB cameras, to map the space and use spatial anchors to synthesize virtual objects based on accurate 3D positional relationships in the real-world 3D space. Drawing is based on 3D spatial recognition, making it an HMD suitable for the purposes of this study. At present, the accuracy of MR alone cannot be expected because the surgeon sets the information with reference to the navigation system, but by adding MR information, the safety of screw insertion and the mental confidence of the surgeon can be achieved by obtaining 3D navigation information while obtaining actual information on bleeding and anatomy in the surgical field. The MR information can be used to obtain actual information on bleeding and anatomy of the surgical field while obtaining 3D information on navigation. So far, it has been reported that, by using MR technology to project intraoperative X-ray fluoroscopic images onto smart glasses, the time spent looking at the image screen was shortened, and the results were good [14]. We have also reported the usefulness of navigation and AR technology applied to microscopic spine surgery [15]. It was possible to perform surgery with more information by utilizing MR, which can display three-dimensional information in the surgical field. Regarding the accuracy of pedicle screw insertion, in the present study, the clinical deviation rate in the M group was 0.83% (intraoperative CT, navigation, MR), which was significantly lower than the clinical deviation rate of 3.1% in the N group (intraoperative CT, navigation). Compared with previous reports, the deviation rate was 11.4% (preoperative CT, navigation) [16], 2.7% (intraoperative CT, navigation) [17], 6.1% (intraoperative CT, navigation, AR) [18], and 2.9% (intraoperative CT, navigation, microscope, AR) [19]. Although the number of cases was small, we consider the use of MR to be useful for improving screw insertion accuracy.

Conventional imaging assistance in spinal surgery requires the surgeon to match the images in the preoperative plan with the actual anatomy during the operation, and it is necessary to spend time and effort while imagining the three-dimensional field in the head, which causes differences in experience and spatial awareness among surgeons. To overcome this, it was necessary to supplement the techniques to ensure accuracy through many years of training.

Although this study did not use navigation, there are reports that preoperative Cobb angles and vertebral height and rotation did not show statistically significant differences in the rate of screw deviation during PS insertion for adolescent idiopathic scoliosis [20], whereas no significant difference was found in vertebral rotation as a factor affecting screw deviation; a high pedicle convergence angle (the angle formed between the vertebral centerline confirmed by CT and the screw trajectory) has been reported [21]. In this study, there was a tendency for vertebral rotation to require further tilting during screw insertion as a factor affecting screw deviation. This means that, to some extent, it is possible to insert screws accurately using navigation for vertebral rotation, but it may be difficult to insert screws accurately even with navigation for cases that require further tilting due to the effects of vertebral rotation. In addition, though both medial and lateral deviations were observed to the same extent when using navigation alone, only lateral deviation was observed when using MRI. Therefore, by confirming the surgical field while also verifying the trajectory confirmed via navigation during screw insertion, it may help to avoid progressing in a direction that could lead to nerve damage by being mindful of changes in the surgical field caused by the pushing force during screw insertion.

With the introduction of this system, it has been shown that spatial virtual line guidance under MR guidance is very useful for improving accuracy and educational effects in spinal surgery. It also allows the surgeon to manipulate the hologram while wearing sterile gloves during surgery, which may reduce the surgeon’s stress during surgery. This is expected to improve the accuracy of surgery and ensure patient safety. In the accuracy evaluation of cervical screw installation using XR by Olexa et al. [22], a phantom model was used, and the trajectory lines of the screws were superimposed and guided through a headset, resulting in all screws being placed in anatomically appropriate positions, with an average error of around 2.73 mm. It has been confirmed that XR technology can support accurate screw installation.

This time, by displaying navigation information in the air above the surgical field, the virtual line guide was able to reduce the need to check the monitor screen of the conventional navigation system, and the surgeon’s eye movement was decreased. This reduces the cognitive load during surgery and suggests that it creates an environment in which the surgeon can concentrate on the procedure.

The use of MR eliminates the need to check conventional monitors, increasing the degree of freedom during surgery. This is also useful for education and training, suggesting that it could be an effective means for multidisciplinary teams to share the same information in real time and hold discussions.

In the future, the technology to accurately align (register) the actual anatomical position of the patient during surgery with the 3D model taken preoperatively is expected as a more precise and reliable navigation tool in cooperation with surgical robots. In a meta-analysis comparing the accuracy and safety of pedicle screw installation with conventional technologies, navigation, robot-assisted, and XR guidance systems, Riewruja found that robot-assisted technology showed the highest accuracy, but did not show significant differences in safety or clinical results [23]. In addition, a systematic review by McCloskey et al. showed that AR technology contributes to improving accuracy and reducing radiation exposure in terms of the effectiveness of XR in surgical education, preoperative planning, and intraoperative guidance in spinal surgery, suggesting that XR may be a game-changer in spinal surgery [24]. Regarding the effectiveness of XR in education, preoperative planning, and intraoperative guides, further clarification of technical requirements and further research are needed.

### 4.1. Limitations

The limitations of this study are the small number of cases and the lack of comparison of differences between surgeons or the lack of random allocation.

In addition, the polygons were aligned manually, and the accuracy of the HMD itself is not guaranteed; therefore, MR cannot be used on its own because it relies solely on existing navigation and is merely a piece of information that can be checked during surgery.

### 4.2. Future Perspective

We propose to use a variety of XR technologies such as HMD to increase certainty in penetration techniques for pedicle screws in spinal surgery, which requires high accuracy. Robot-assisted spinal surgery has been introduced in recent years, but the problem is that the cost will increase. It is possible to reduce costs by using XR technology instead of robots, but the accuracy of XR technology is important in this case.

In the future, we will increase the number of cases and compare the XR combination group with the conventional method that does not use XR technology to clarify the usefulness of XR. Ultimately, advances in XR systems will enable them to demonstrate accuracy equal to or greater than that of current navigation systems, replacing navigation systems and promoting significant cost reductions and accurate surgical techniques.

There are several challenges in the introduction of XR technology surgical support in Japan. First, in order for the surgeon to be proficient in handling XR technology, new training that differs from that with conventional technology is required. In addition, it has been pointed out that there are problems with the physical burden of wearing HMDs and fatigue associated with their prolonged use. In recent years, HMDs have been improved in terms of weight reduction, higher screen resolution, higher definition, improved frame rate, and reduced shaking due to higher accuracy of position sensors, and the stress of conventional wearers has been decreased to some degree. Furthermore, in Japan, the introduction of new technologies requires careful consideration and sufficient clinical trials to prioritize patient safety.

## 5. Conclusions

Virtual lines with MR technical support in navigational spinal surgery can obtain good pedicle screw insertion accuracy by presenting navigation system information three-dimensionally in the surgical field. It was also confirmed that this showed excellent operability during surgery. Future studies need to increase the number of cases and verify their long-term usefulness and safety. This system is expected to become a standard support tool for spinal surgery, improve the success rate of surgery, and reduce the burden on the surgeon.

## Figures and Tables

**Table 1:** Number of screws in the Rao classifications for the M group and the N group and results for clinical deviation (Grade 2 + Grade 3).

	M Group (361 Screws)	N Group (391 Screws)
Grade 0	333 (92.2%)	355 (90.8%)
Grade 1	25 (6.9%)	24 (6.1%)
Grade 2	1 (0.3%)	11 (2.8%)
Grade 3	2 (0.6%)	1 (0.3%)
Clinical deviation	3 (0.83%)	12 (3.1%)

**Table 2:** Pedicle size by grade and group.

		Grade 0/1			Grade 2/3			*p*-Value (Grade 0/1 vs. Grade 2/3)
		Estimated Average Value	95% Confidence Interval		Estimated Average Value	95% Confidence Interval		
			Lower Limit	Upper Limit		Lower Limit	Upper Limit	
**●** **Overall** **(M group + N group)**								
	Pedicle size	3.61	3.34	3.87	2.91	2.14	3.69	0.066
**●** **M group**								
	Pedicle size	3.61	3.18	4.05	2.14	0.51	3.78	0.071
**●** **N group**								
	Pedicle size	3.61	3.28	3.95	3.09	2.19	3.99	0.239

Data display: estimated mean [95% confidence interval]. *p*-value: linear mixed model (fixed effects: time (preoperative/intraoperative) and group (individual vertebrae or combined vertebrae); random effects: ID). Compared with grades 0 and 1, grades 2 and 3 tended to have smaller pedicle diameters, but the difference was not significant.

**Table 3:** Changes in vertebral rotation between preoperative conscious prone CT and intraoperative anesthetic prone CT in the M and N groups combined.

Group	Preoperative			Intraoperative			*p*-Value (Preoperative vs. Intraoperative)
	Estimated Average Value	95% Confidence Interval		Estimated Average Value	95% Confidence Interval		
		Lower Limit	Upper Limit		Lower Limit	Upper Limit	
Th1	7.70	−7.71	23.11	3.70	−11.71	19.11	0.711
Th2	8.53	2.56	14.51	5.25	−0.72	11.22	0.420
Th3	10.11	4.49	15.72	9.48	3.87	15.09	0.870
Th4	8.68	4.57	12.80	9.93	5.82	14.05	0.643
Th5	5.27	2.00	8.54	9.02	5.75	12.29	0.066
Th6	2.65	−0.49	5.79	6.29	3.19	9.39	0.058
Th7	−1.93	−5.03	1.18	2.32	−0.78	5.43	**0.026**
Th8	−4.65	−7.75	−1.54	−0.12	−3.22	2.99	**0.018**
Th9	−6.46	−9.53	−3.39	−2.06	−5.13	1.00	**0.019**
Th10	−6.43	−9.26	−3.61	−3.02	−5.84	−0.19	**0.043**
Th11	−2.15	−4.93	0.62	−1.60	−4.37	1.18	0.734
Th12	1.78	−0.97	4.53	1.33	−1.43	4.08	0.780
L1	4.62	1.72	7.53	3.18	0.27	6.08	0.408
L2	7.26	3.95	10.58	2.45	−0.86	5.76	**0.020**
L3	6.07	2.60	9.54	0.78	−2.70	4.25	**0.016**
L4	−0.20	−6.20	5.80	−5.06	−11.06	0.94	0.233

Data display: Estimated mean [95% confidence interval]. *p*-value: linear mixed model (fixed effects: time (preoperative/intraoperative) and group (individual vertebrae or combined vertebrae); random effects: ID). Significant differences were observed in the mid-thoracic and lumbar spine before and after surgery. The accuracy of the screw insertion angle planned on preoperative CT was not high.

**Table 4:** Insertion angle by grade and group.

		Grade 0/1			Grade 2/3			*p*-Value (Grade 0/1 vs. Grade 2/3)
		Estimated Average Value	95% Confidence Interval		Estimated Average Value	95% Confidence Interval		
			Lower Limit	Upper Limit		Lower Limit	Upper Limit	
**●** **Overall** **(M group + N group)**								
	Angle	2.09	0.90	3.28	6.01	1.82	10.21	0.061
**●** **M group**								
	Angle	4.67	2.98	6.37	12.74	4.75	20.74	**0.046**
**●** **N group**								
	Angle	−0.70	−1.57	0.18	2.42	−2.47	7.31	0.218

Data display: estimated mean [95% confidence interval]. *p*-value: linear mixed model (fixed effects: time (preoperative/postoperative) and group (individual vertebrae or combined vertebrae); random effects: ID). In the M group, Grade 2/3 vertebral rotation resulted in a tendency for screws to shift, requiring insertion at a stronger oblique angle than usual, whereas no significant difference was observed in the N group.

## Data Availability

The original contributions presented in this study are included in the article. Further inquiries can be directed to the corresponding author.
